# Effects of bottom-up and top-down attentional processes on change blindness for COVID-related stimuli: influence of heart rate variability

**DOI:** 10.3389/fnins.2024.1458627

**Published:** 2024-11-22

**Authors:** Francesca Favieri, Giovanna Troisi, Giuseppe Forte, Ilaria Corbo, Giulia Marselli, Barbara Blasutto, Renato Ponce, Enrico Di Pace, Viviana Langher, Renata Tambelli, Maria Casagrande

**Affiliations:** ^1^Department of Dynamic and Clinical Psychology and Health Studies, “Sapienza” University of Rome, Rome, Italy; ^2^Department of Psychology, “Sapienza” University of Rome, Rome, Italy; ^3^Department of Experimental Psychology, and Mind, Brain, and Behavior Research Center (CIMCYC), University of Granada, Granada, Spain

**Keywords:** change blindness, attention, flicker task, emotional stimuli, COVID-related stimuli, heart rate variability

## Abstract

**Introduction:**

Top-down mechanisms that regulate attentional control are influenced by task demands and individuals’ goals, while bottom-up processes are influenced by salient stimuli. Analogous networks are involved in both processes (e.g., frontostriatal areas). However, they are affected differently by the emotional salience of stimuli, which determines the allocation of attention. This study aims to determine whether the recent pandemic experience continues to exert an influence on cognitive processes. To this end, the study will determine attentional biases toward pandemic-related stimuli compared to negative and neutral stimuli. Furthermore, the study will investigate whether pandemic-related stimuli influence top-down and bottom-up attentional processes and whether the latter affect autonomic control as indexed by Heart Rate Variability (HRV).

**Methods:**

Ninety-six undergraduate students completed a Flicker Task with stimuli categorized by emotional valence (neutral, negative non-COVID, negative COVID-related). This paradigm involves the presentation of two different pictures, which are identical except for a specific detail. The task required to detect the specific detail that has been changed. Given that the task employs images of natural scenes, participants tend to focus more on specific areas of the scene than others. As a result, changes in central interest (CI) areas are detected more rapidly than changes in marginal interest (MI) areas. Participants’ response times (RTs) at the task and their HRV data were used to assess attentional performance and the associated autonomic nervous system activity.

**Results:**

The results indicate slower responses to COVID-related stimuli than negative and neutral stimuli for both CI and MI changes, requiring the involvement of bottom-up (CI changes) and top-down (MI changes) processes. The HRV was associated with a slower detection of CI changes in COVID-related scenes.

**Discussion:**

These findings highlight the intricate interplay between emotional salience, attentional mechanisms, and physiological responses to threatening stimuli. Contextual factors, particularly those related to pandemic-related stress, influence attentional processing and its relationship with autonomic activity.

## 1 Introduction

The world around us is rich in elements that make simultaneous and detailed analysis impossible, despite the human being need to contextualize information for the purpose of survival. The visual scene must be carefully explored by the eyes, especially in emergency situations (e.g., danger, achieving a relevant goal) in which rapid interaction with the environment is required (Benicasa et al., 2012; Bendall and Thompson, 2015). To optimize the search procedure and visual attention and to allow task success, two different processes involving multiple brain networks are active, i.e., top-down and bottom-up attentive mechanisms. Both processes can be considered two different components of attention. The first, which is driven by the individual’s control, involves multiple cognitive dimensions (including attention and memory) and is characterized by the allocation of attention to items that match target features inhibiting environmental distractors (Schneider and Shiffrin, 1977) in a goal-directed frame. Consequently, top-down processes are task-dependent and entail eye movements in search of a particular object when visual attention is involved. In contrast, the bottom-up mechanisms reflect the automatic capture of attention by salient information, regardless of task demand (e.g., Itti and Koch, 2001). These last mechanisms are influenced by the exogenous properties of the stimuli and are generated by combining information from the retina and primary visual cortex regions.

The complex relationship between these two distinct types of processes was investigated using two main frameworks: (i) the shared neural substrate involving the frontoparietal network (for a review, see Katsuki and Constantinidis, 2014) and (ii) the role of emotional salience on the bottom-up and top-down elaboration of stimuli (Mohanty and Sussman, 2013). Considering bottom-up processing, empirical evidence has demonstrated that attention is involuntarily drawn toward emotional stimuli in neutral scenes (Carretié, 2014). This phenomenon is observed to be only partially modulated by frontoparietal areas (Vuilleumier and Driver, 2007). In top-down mechanisms, the role of motivational factors that alter the degree to which emotional stimuli capture attention attention has been widely confirmed (Kristjánsson et al., 2010). A recent study by Mrkva et al. (2019) highlighted that the perceived emotional intensity of a stimulus can be influenced and amplified by top-down attentional mechanisms. This suggests that voluntary processes can interfere with the bottom-up elaboration of a scene. Given these considerations, the dichotomous theorization of top-down and bottom-up mechanisms is unsuitable for detecting processes beyond visual attentional processing.

Another research branch that would furnish interesting insight into the nature and interplay of bottom-up and top-down attentional processes would involve monitoring the physiological state of individuals during visual attentional elaboration (Kim and Anderson, 2021). This perspective would help in understanding the emotional role of the stimuli in the modulation of distinct attentional mechanisms. The neurovisceral integration model (Thayer and Lane, 2000) posits that a neural network connects the autonomic, emotional, and cognitive self-regulation processes, which may provide a means of understanding the emotional activation driving the bottom-up and top-down features of attention. One indirect index of the functionality of this network is the heart rate variability (HRV), which is an expression of the cardiac vagal tone. Research indicates that higher resting-HRV is associated with more adaptive top-down and bottom-up cognitive modulation of emotional stimuli (e.g., Laborde et al., 2017; Forte et al., 2021; Gross, 2014). In contrast, lower resting HRV has been linked to hypervigilant and maladaptive cognitive responses to emotional stimuli. With the aim to investigate both the automatic (bottom-up) and voluntary (top-down) components of attention, a reliable experimental paradigm is the change detection flicker tasks (Kamkar et al., 2018). This paradigm (Rensink et al., 1997) involves the presentation of two different pictures, which are identical except for a specific detail. The pictures are presented in a repetitive sequence, with each image separated by a brief gray screen. The observers are required to search the scene for the specific detail that has been changed between the two pictures (A → A′) until they identify it. The change can be in different areas of the visual scenes. Focused attention, involved in the flicker paradigm, moves in the environment to overcome the change blindness phenomenon, characterized by a difficulty in localize and detect changes at specific positions on the retina (Simons and Levin, 1997). Given that the task employs images of ecological scenes, participants tend to accord greater attention to specific areas of the scene than to others. Consequently, changes in objects of central interest (CI) are detected more rapidly than changes in marginal-interest (MI) objects (Rensink et al., 1997). The detection of these changes may require the involvement of different attentional processes, depending on their degree of salience and type of interest. Bottom-up attentional processes may be engaged in the detection of CI changes, while top-down processes may be involved in detecting changes of MI (Wright, 2005; Maccari et al., 2013; Favieri et al., 2020; Forte et al., 2021). The inclusion of pictures and manipulations that can activate both automatic and voluntary attentional processes in a single task, allows for an independent analysis of the detection times of scene changes (CI; MI). This makes the task valuable and unique for the analysis of the complexity of the attentional system. For this reason, this change detection paradigm was adopted with different stimuli, such as those related to addiction, food, or phobia (Jones et al., 2003; Favieri et al., 2020; McGlynn et al., 2008), as well as in different clinical conditions involving attentive processes (such as ADHD; Maccari et al., 2013, or anxiety; Forte et al., 2021). These investigations have yielded interesting insight into the attentional features associated with the processing of salient or threatened stimuli (e.g., Maccari et al., 2014). Moreover, a study indicates a correlation between HRV and performance on the Flicker task, which suggests that the neurovisceral integrative network plays a predictive role in attentional mechanisms toward emotionally activating scenes (Forte et al., 2021).

According to this framework, this study aimed to investigate the bottom-up and top-down attentive elaboration of salient stimuli further, with a focus on the role of vagal-mediated HRV. Specifically, given the modulator effect of emotional salience in biasing attention (Cisler et al., 2011; Mrkva et al., 2019; Olatunji et al., 2013), our objective was to focus on the nature of the scenes, differentiating different real-life experiences that may affect our ability to detect the environment in multiple ways. To achieve this aim, we focused on the recent pandemic experience. The pervasive influence of the media in recent decades has led to the exposure of the global population, regardless of cultural background, to a growing array of stimuli perceived as threatening (e.g., war, global warming, natural disasters). In the last years, the prevalence of these threatening stimuli has been further compounded by the unprecedented dissemination of information related to the COVID-19 pandemic. During the pandemic, the frequent and vivid presentation of information about the virus and its effects on health and social contexts captures attention automatically (Cannito et al., 2020). Consequently, individuals were found to have difficulty diverting their attention from COVID-19-related stimuli, resulting in an effect known as attentional bias. Moreover, individuals’ concerns and anxiety levels dramatically increased during the pandemic (Casagrande et al., 2020, 2021; Forte et al., 2020a, 2020b; Favieri et al., 2021a; Tambelli et al., 2021), which would have influenced the focus of attention on information related to the pandemic, reinforcing fear and concern via top-down processing (Jun et al., 2021; Sisk et al., 2022). Many studies in this field has explored the impact of traumatic experience on attentional bias, such as in the case of post-traumatic symptomatology (Elsesser et al., 2004; Olatunji et al., 2013; Olatunji et al., 2022). For example, threatening stimuli PTSD-related have been observed to impair task performance on the Emotional Stroop Task in a sample of patients with PTSD (Cisler et al., 2011). Moreover, some studies reported that target detection is affected by the emotional salience, which is affected by personal life experiences (Wingenfeld et al., 2006; Olatunji et al., 2013; Olatunji et al., 2022). Accordingly, the use of salient stimuli that are linked to the real-life experience represents an effective method for evaluating the role of emotional reactivity (to both threatening and non-threatening stimuli) in the attentive elaboration of a scene.

However, to the best of our knowledge, no study has investigated the top-down and bottom-up processes in attentional bias related to the COVID-19 pandemic. Furthermore there is a scarcity of literature that has directly compared the effect of stimuli related to the pandemic to the effect of other threatening stimuli, such as those related to grief, war, or violence. A recent study (Santacroce and Tamber-Rosenau, 2024) examined the distinction between conventional emotional distractors (positive, negative and neutral stimuli) and the distractive effect of stimuli related to community crises (i.e., stimuli from the Harvey Hurricane and pandemic-related stimuli associated with the COVID-19 pandemic). Two experiments were conducted: the first utilized a picture emotional attentional blink task (Experiment 1: Harvey Hurricane) while the second employed a word emotional attentional blink task (Experiment 2: COVID pandemic). The results showed that the emotional distractors impeded the subsequent detection of targets in the context of rapid-flowing stimuli. The authors observed that the distracting effect was more pronounced for conventional distractors, suggesting that crises that impact communities, such as the pandemic experience, may not necessarily influence attentional activity through emotional reactivity to crisis-related stimuli. Our study aimed to further investigate the role of emotional salience on attentional processing of environmental stimuli with a particular focus on the distinction between automatic and voluntary attentional elaboration.

Therefore, we hypothesized that there would be different patterns in both the physiological (HRV) and cognitive responses to emotional stimuli related to the pandemic and non-COVID related stimuli, due to the different cognitive evaluation of negative stimuli of different nature. Moreover, we expected that the analysis of attentional responses via the adoption of the Emotional Flicker Paradigm would yield further insights. This objective would help us to explain how the pandemic experience has affected our attentional process in both automatic and voluntary features.

## 2 Materials and methods

### 2.1 Participants

Ninety-six undergraduate students were recruited at the University of Rome “Sapienza.” Only participants who met the following inclusion criteria were included in the study: (i) age between 18 and 35 years; (ii) absence of severe chronic medical (e.g., cardiovascular disorders) or psychiatric (e.g., schizophrenia) conditions; (iii) never having suffered a traumatic brain injury or heart stroke; (iv) having lived in Italy during the lockdown; (iv) good visual acuity. The exclusion criteria were investigated during the anamnestic interview at the beginning of the experimental session. All participants signed a written informed consent form before the experimental procedure.

### 2.2 Measures

#### 2.2.1 Sociodemographic variables

A semi-structured interview was conducted in order to collect the following sociodemographic data: (i) age and educational level; (iii) potential exclusion criteria (i.e., traumatic brain injury, heart stroke, and severe chronic diseases); (iii) presence of medical or psychiatric conditions; (iv) potential pharmacological therapies; and (v) habits related to cigarette consumption.

#### 2.2.2 Change blindness: emotional flicker task

According to the emotional valence of the stimuli (i.e., Neutral; Negative, COVID-related), three blocks of the emotional Flicker Task were administered (see general procedure for details).

##### 2.2.2.1 Apparatus

The Flicker Task was administered on a Personal Computer with a 19-inch high-definition monitor. The administration of stimuli and response time recordings were programmed using E-Prime 2.0 software on an Intel Core i5 PC. Responses were given on the computer keyboard.

##### 2.2.2.2 Stimuli

For the neutral and negative conditions, sixteen pictures from the IAPS (International Affective Picture System; Lang et al., 2008) were selected according to their emotional valence: eight pictures (9,253, 9,433, 3,500, 2,205, 9,410, 3,530, 9,254, 6,520) with negative valence (mean valence score = 1.92 ± 0.20; mean arousal score = 6.18 ± 0.87), eight pictures (7,550, 2,102, 7,036, 2026, 7,130, 5,471, 2,411, 2,308) with neutral valence (mean valence score = 4.99 ± 0.25; mean arousal score = 3.47 ± 0.31).

To validate the negative COVID-related stimuli, an independent sample of 43 participants (67% females; mean age = 39,56, SD = 13,49, age range = 20–71) evaluated a pool of 21 pictures covid-related, which were previously selected from online databases free of charge or copyright. Participants were required to rate the emotional negative valence on a numerical rating scale (i.e., from 1 = “extremely negative” to 10 = “extremely positive”). The arousal associated with the pandemic experience elicited by the pictures was assessed by a numeric scale indicating the grade of association of the pictures with the COVID pandemic (i.e., from 1 = “not related to the pandemic” to 10 = “extremely related to the pandemic”). Following the validation of the COVID-related stimuli, eight pictures were selected from the original set of stimuli (mean valence score = 1.62 ± 0.52, mean arousal score = 9.51 ± 0.11) according to their negative valence and COVID-related arousal.

The pictures (640 × 480 pixels) were manipulated by using Adobe Photoshop software (version CS6-13.0). Two versions of each picture were generated, differing only in the presence or absence of a single detail (49 × 49 pixels).

**Figure 1:**
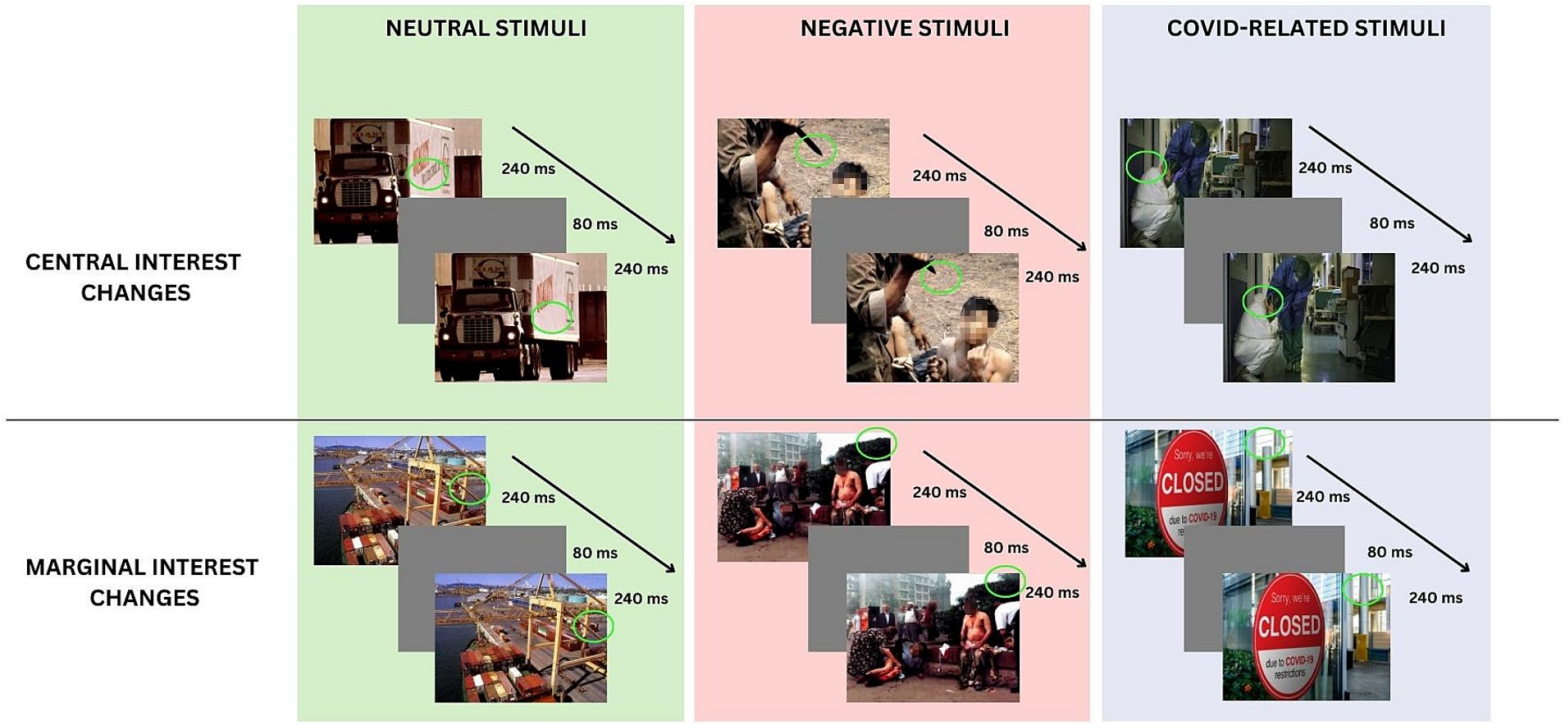
Flicker task procedure and example for each condition.

In accordance with the procedure indicated by Rensink et al. (1997) and followed by previous studies adopting this paradigm (e.g., Forte et al., 2021), half of the pictures for each valence exhibited a change of central interest (CI), and half of the pictures exhibited a change of marginal interest (MI) (see Figure 1). The Negative and Neutral stimuli were derived from the same set of images used and validated in previous studies (see Maccari et al., 2014; Forte et al., 2021). Figure 1 shows the Flicker Procedure and some examples of the stimuli. To insert changes in MI and CI in the COVID-scenes, an independent group of 20 undergraduate students (mean age 23.01; SD = 2.15) was recruited. Following the observation of each picture for 3 s, participants were required to write all the elements of the picture that they could recall on one grid in the location that they remembered. According to the standard procedure (Rensink et al., 1997), the items mentioned by more than 90% of the observers were of CI; the items written by no more than two participants were defined as MI. Pictures adopted in the studies are shared in OSF.^1^

##### 2.2.2.3 Procedure

Participants were seated approximately 56 cm from the computer screen and tested individually in a quiet, dimly illuminated room. On each trial, an original and a modified version of a picture alternated repeatedly (240-ms display time), separated by a gray screen (80 ms), until the participant detected the change. An example of the procedure is shown in Figure 1.

Participants were instructed to press the spacebar on the keyboard as soon as they detected the change and then to describe the change verbally by typing the changed element on the computer keyboard. At the beginning of the task, three practice trials were proposed. Each participant underwent three blocks of the Flicker task with different emotional valence (i.e., neutral, negative non-COVID, negative COVID-related). Response times (RTs; ms) and Accuracy (number of correct responses) were collected, and according to the procedure proposed by Maccari et al. (2013) and adopted in previous studies (Forte et al., 2021; Favieri et al., 2020), RTs in trials where participants did not detect the change were replaced by the mean RTs + 2.5 SD for that condition. All participants showed a percentage of accuracy greater than 50%.

#### 2.2.3 Physiological assessment

Heart Rate Variability: HRV was recorded using the Firstbeat Bodyguard-2 (Firstbeat Analytics, Jyvaskyla, Finland). Kubios HRV Analysis 3.4.3 software was used to process the signals. Both frequency and time domain of the HRV were considered. The frequency-domain analysis provided the mean spectral power measures of the Low Frequency (LF-HRV, 0.04–0.15 Hz) and High-Frequency (HF-HRV, 0.15–0.4 Hz) bands; while LF reflect the influence of both sympathetic and parasympathetic activity, the HF indicates changes in vagal control of the heart. The time-domain analysis yielded the standard deviation of the mean RR interval (SDNN), which indicates the variability in the recording period, and the root mean square of the successive standard deviation (RMSSD), an index of the vagal tone. In accordance with the guidelines for the correct assessment of HRV (Laborde et al., 2017), participants were informed to avoid smoking, eating, and drinking beverages containing caffeine or theine. HRV was assessed for 5 min at rest (i.e., baseline condition), during the three blocks of the Flicker Task, and at the end of the experimental session. According to previous studies, all the indices were transformed into natural logarithms for statistical analyses (e.g., Forte et al., 2023).

### 2.3 General procedure

The entire study protocol was approved by the Ethics Committee for Transdisciplinary Research of Sapienza University of Rome (Resolution No. 87/2023) in accordance with the ethical guidelines of the Declaration of Helsinki. The experimental procedure took place in a quiet room at the University of Rome “Sapienza.” Each participant was tested individually in a single experimental session. At the beginning of the session, the written informed consent was signed, the anamnestic socio-demographic interview was conducted. Subsequently, the ECG recording device was positioned. Participants were instructed to relax for 5 min to register resting HRV (baseline). In order to record HRV data for each emotional condition, three blocks of the Flicker paradigm (Neutral; Negative, COVID-related) were performed in a between-subject balanced order (e.g., subj1 = Neutral; Negative, COVID-related; Subj2 = Negative, Neutral; COVID-related; Subj3 = Neutral; COVID-related; Negative). Each block presented the pictures with CI and MI in a randomized order. Finally, the device for HRV assessment was removed.

### 2.4 Data analysis

The statistical analyses were performed using the Jasp software. Participants’ performance in each block of the Flicker task was indexed by their response times (RTs), which were corrected according to the proportion of accuracy (difficulty index; Rezigalla et al., 2024) by using the formula Response Time/(number of correct items/total items for each condition), helping in reducing the high variability of RT. A within-model analysis of variance (ANOVA) was performed considering the Type of change (CI and MI) and the Valence (neutral, negative, COVID-related) as the independent variables. The RTs corrected for accuracy were considered as the dependent variable. To analyze the significant interaction effects of the statistical design, planned comparisons were adopted. Furthermore, Pearson’s correlations were performed to evaluate the relationship between performance (RTs) and HRV during both the baseline and the Flicker task. Given the high number of correlations and comparisons, the *p*-values adjusted using Bonferroni’s correction and significant effects were set at *p* < 0.01.

## 3 Results

### 3.1 Descriptive statistics

The study sample consisted of 96 individuals, 56 of whom were female and 40 of whom were male (mean age = 24.05, SD = 3.02, range = 19–35). Descriptive statistics about the socio-demographic characteristics of the sample, the HRV indices and the mean RTs are reported in Table 1.

**Table 1:** Descriptive statistics of socio-demographic data and HRV indices.

N = 96	Mean (SD)
Socio-demographic data	
Sex (F/M)	56/40
Age	24.05 (3.02)
Education	17.55 (2.10)

	Baseline	COVID-related	Neutral	Negative
Heart rate variability indices				
SDNN	3.67 (0.39)	3.76 (0.35)	3.75 (0.32)	3.80 (0.33)
RMSSD	3.31 (0.53)	3.47 (0.49)	3.49 (0.46)	3.52 (0.47)
LF	6.78 (0.86)	6.96 (0.73)	6.93 (0.73)	7.00 (0.74)
HF	5.74 (1.09)	6.03 (1.07)	5.98 (1.02)	6.09 (1.04)

SD, standard deviation; SDNN, standard deviation of all normal RR (NN) intervals; RMSSD, root mean square of successive differences; LF, low frequencies; HF, high frequencies. HRV indices are transformed in natural logarithms.

### 3.2 ANOVA results

The main effects of the Type of change (F_1,85_ = 169.69; *p*< 0.001; η2 = 0.16,) and Valence (F_2,170_ = 33.59; *p* < 0.001; η2 = 0.13) were statistically significant. The RTs were significantly faster for CI changes compared to MI changes (*t* = −13.03; *p* < 0.001). Furthermore, the RTs were significantly slower for COVID-related stimuli compared to both negative (*t* = 7.45; *p* < 0.001) and neutral stimuli (*t* = 6.69; *p* < 0.001). The difference between the negative and neutral stimuli was not statistically significant (t < 1).

**Figure 2:**
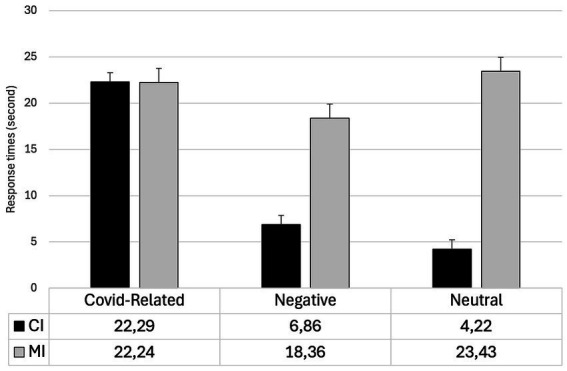
Means and St. errors of the RTs considering interaction type for change x valence.

The interaction Type for change x Valence was significant (F_2,170_ = 65.32; *p* < 0.001; η2 = 0.13). Planned comparisons revealed that the CI changes were detected significantly slower when COVID-related stimuli were employed compared to when negative (*t* = 10.24; *p* < 0.001) and neutral stimuli (*t* = 11.94; *p* < 0.001) were used. Differently, RTs were faster for negative stimuli than for neutral stimuli, although this difference was only marginally significant (*t* = −2.95; *p* = 0.052). No significant differences in MI changes were observed between negative and COVID-related stimuli (*t* = −1.98; *p* = 0.73). Furthermore, the differences between CI and MI changes were significant for both negative (*t* = −9.01; *p* < 0.001) and neutral stimuli (*t* = −14.75; *p* < 0.001) but not for COVID-related stimuli (*t* = 1.22; *p* = 0.22). For each block of stimuli, mean RTs and standard deviations for both CI an MI changes are reported in Table 2. The Type of change x Valence interaction is reported in Figure 2.

**Table 2:** Mean (± SD) of response times for changes in central interest (CI) and marginal interest (MI) areas for COVID-related, negative, and neutral stimuli.

	COVID-related stimuli	Neutral stimuli	Negative stimuli
CI	22.29 (17.02)	4.22 (2.71)	6.86 (3.15)
MI	22.24 (15.30)	23.43 (12.38)	18.36 (6.77)

Response Times (RTs) are corrected for the proportion of accuracy. CI, changes of central interest; MI, changes of marginal interest.

### 3.3 Pearson’s correlations

Pearson’s correlations were performed separately for each of the three blocks of the Flicker Task in order to investigate the relationship between HRV indices and the changes detected for each type of emotional stimulus. In the Table 3 are reported all the correlations adjusted by Bonferroni’s correction.

**Table 3:** Pearson’s correlations between RTs and HRV indices for changes in central interest (CI) and marginal interest (MI) areas for COVID-related, negative, and neutral stimuli.

	HRV during baseline				HRV during flicker task			
	SDNN	RMSSD	LF	HF	SDNN	RMSSD	LF	HF
COVID-related								
CI	0.19	0.22	0.12	0.20	0.27*	0.23	0.26*	0.24
MI	0.06	0.12	−0.05	0.08	0.13	0.11	0.13	0.11
Neutral								
CI	0.03	0.05	−0.02	0.08	0.21	0.17	0.19	0.15
MI	0.10	0.04	0.02	0.03	0.04	0.16	−0.01	0.08
Negative								
CI	0.05	−0.02	0.06	−0.01	−0.04	−0.002	−0.01	−0.04
MI	−0.10	−0.07	−0.05	−0.02	−0.16	−0.11	−0.09	−0.03

Response Times (RTs) are corrected for the proportion of accuracy. CI: changes of central interest; MI: changes of marginal interest. SDNN: standard deviation of the mean RR interval; RMSSD: root mean square of the successive standard deviation; LF: Low- Frequency HRV; HF: High-Frequency HRV; *p < 0.01.

Pearson’s correlations were performed to investigate the association between HRV indices and RTs in both CI and MI changes assessed during both the baseline and reactivity (i.e., the COVID-related trials of the Flicker Task). The results of the correlation analyses between performance for CI changes and the HRV indices indicate positive and significant correlations between RTs and the following HRV indices: SDNN (*r* = 0.27; *p* < 0.01) and LF (*r* = 0.26; *p* = 0.01) registered during the task performance, indicating that slower responses in detecting CI changes were associated with higher HRV indices. No other significant correlation was reported for both CI and MI changes in reactivity or resting recording of HRV. All values are reported in Table 3.

No correlation was found between HRV and change detection for neutral stimuli (*p* > 0.05). All values are reported in Table 3.

For both CI and MI changes, the results did not reveal significant correlations between HRV indices and RTs (all p > 0.05). All values are reported in Table 3.

## 4 Discussion

The primary objective of this study was to examine the bottom-up and top-down attentive processes involved in the detection of emotionally salient scenes. Specifically, the study aimed to identify the physiological and cognitive differences during the detection of changes in COVID-related and non-COVID stimuli with the same negative valence in the non-clinical population.

The most intriguing aspect emerging by this study is that both automatic and voluntary elaboration of pictures showing COVID-related scenes is characterized by an overall slowness. In the Flicker paradigm, it is well known that individuals report faster detection times when changes occur in central interest areas of the scene than when they occur in marginal interest areas (Rensink, 2002; Simons, 2000). This effect is robust and has been interpreted as resulting from a pop-out effect of changes occurring in areas of central interest, which led to an automatic capture of attention (Maccari et al., 2013, 2014; Favieri et al., 2021a,b).

Considering the emotional valence, it can be observed stimuli that elicit fear or negative emotions tend to capture attention more effectively than neutral stimuli. Therefore, due to evolutionary processes related to survival, they are faster detected (Öhman and Mineka, 2001). However, in our previous studies employing the Emotional Flicker Paradigm, we have observed a faster change detection of negative than neutral scenes in attentional top-down processing but not in bottom-up elaboration (Forte et al., 2021; Maccari et al., 2014). The results of the present study did not confirm this first evidence. However, the scenario changes when we consider the COVID-related stimuli. These stimuli are highly negative, as evidenced by the results of the pictures evaluation procedure, which indicated a high level of negative valence for both negative IAPS stimuli and COVID-related ones. However, while the response to negative stimuli is characterized by faster detection of changes in central interest areas, no differences were observed in the detection time changes in central and marginal interest areas for COVID-related stimuli. How can we justify this result? The negative salience of COVID-related scenes may be ascribed to the fact that they evoke memories of the recent pandemic experience. It seems plausible that this detection pattern may be explained by the high emotional and cognitive load associated with pandemic-related information. It is possible that concerns related to the pandemic may give rise to general anxiety rather than specific fears. Consequently, the negative valence of the COVID-related stimuli may be elaborated in a manner that differs from the negative non-COVID stimuli included in this study, which represented scenes of war and violence (Smith et al., 2022). This hypothesis is corroborated by the findings of studies indicating that the relationship between anxiety and attention is less straightforward than that between fear and attention (Robinson et al., 2013a, 2013b). In fact, patients with anxiety disorders exhibit deficits in brain regions regulating cognitive control even when they do not always perform more poorly on cognitive control tasks, with heterogeneous outcomes (Eysenck et al., 2007; Lagarde et al., 2010).

Another interesting interpretation would be supported by studies on attentional bias (e.g., Ho and Mussap, 2020; McCarthy and Reed, 2024). The results of these studies suggest that when individuals are presented with negative stimuli non-COVID related, their attentional resources are directed to the relevant areas activating the pop-up effect. This would be ascribed to a defensive strategy of the attentional system, which guarantees the faster detection of potential threatening elements (e.g., weapon, blood) via an automatic and low energy process. Conversely, for COVID-related scenes, attention is diffuse for the entire image, due to its globally threatening nature and the pandemic-related implications. Consequently, this would affect the performance of automatic attentional processing by disturbing the pop-up effect of salient elements in the scene.

Also, studies on attentional bias suggest the existence of two distinct phases of attention elaboration in individuals experiencing various forms of psychological distress (e.g., PTSD, trauma exposure, anxiety) (Albery et al., 2021). The initial phase is characterized by heightened vigilance towards stimuli perceived as threatening, which is followed by a second phase marked by a threat-avoidance attentional pattern (for a meta-analysis, see Bar-Haim et al., 2007). Consistently, when individuals process pandemic-related stimuli, which are perceived as threatening due to their traumatic impact (Cannito et al., 2020) they are likely to exhibit both immediate vigilance and a slower disengagement from the threat source in the secondary phase (Albery et al., 2021). This affects overall attentional processes. This may be attributed to the distinctive and pervasive impact of the pandemic on cognitive processing and, subsequently, the representation of those contexts (Lee and Lee, 2023; Favieri et al., 2021a,b). In this regard, the outcome of the physiological recording may provide further insight. HRV is often used as an indicator of autonomic nervous system flexibility and stress resilience (Thayer et al., 2012). According to Thayer et al. (2009), higher HRV has been associated with enhanced top-down attentional control, which can be considered an index of the integrity of the brain networks involved in cognitive and emotional regulation (Thayer et al., 2009; Thayer and Lane, 2000). In this sense, a flexible autonomic nervous system as indicated by high HRV facilitates the efficient allocation of attentional resources in a top-down manner. However, the results of this study did not corroborate the aforementioned hypothesis, as no significant correlation was observed between HRV indices and performance in the Flicker task during the top-down detection process (changes of marginal interest) regardless of the type of stimuli and their salience. Nevertheless, we found that low frequency of HRV and standard deviation of the mean RR interval, recorded during the task, were positively correlated with the detection of central interest changes of COVID-related stimuli. This correlation suggests the role of the vagal tone in modulating bottom-up attentional processes and suggest an association between an increasing of the cardiac activity during the task and lower cognitive functioning (Hilgarter et al., 2021). Since previous studies have reported a sensitivity of low frequency of HRV and standard deviation of the mean RR interval, to changes in arousal (Bulut et al., 2018; Laborde et al., 2017), it can be assumed that attentional process, specifically its bottom-up dimension, may be influenced by hyperarousal generated by the nature of stimuli, which is thought to reflect a shift toward sympathetic dominance and a heightened state of arousal (Porges, 2007). The higher HRV associated with poorer performance observed in our study may indicate an inappropriate reaction to the sustained stress of the pandemic context. This is consistent with recent findings, suggesting that chronic stress can result in altered autonomic responses and cognitive impairment (Kim and Anderson, 2021). Despite these interesting findings, as suggested by Park and Thayer (2014), evidence substantiating the correlation between HRV and both bottom-up and top-down attention remains scarce, and further studies are needed to investigate the cardiac autonomic trend during tasks involving emotional stimuli of different nature (as for COVID vs. non-COVID negative stimuli).

Considering both behavioral and physiological results of our study, the nature of the stimuli appear to be relevant in the results and are consistent with those of McRae et al. (2012), who proposed an interesting thesis that can be utilized to interpret our results. The authors focused on the role of physical characteristics of the stimuli in providing emotional information during the bottom-up process. It was demonstrated that the emotional response is deactivated when perceptual inputs are no longer present. Accordingly, to evaluate the scene, the emotional meaning of the stimulus must be translated into a linguistic representation. Therefore, the top-down process occurs and interacts with the automatic one. In this context, semantic networks represent learned experiences, emotional and cognitive situations, and contexts that influence the ability to detect the environment and its changes (McRae et al., 2012).

Considering this evidence, it can be postulated that when COVID-related stimuli must be elaborated, top-down processes may have an interferential effect during the detection of central interest changes. This may result in a reduction of the difference in change detection between central and marginal interest areas, which could impair bottom-up processes. The retrospective evaluation of the participants’ emotions about the pandemic experience, as reported by participants in this study, indicates that the pandemic is still perceived as negative. This evaluation is consistent with the current research findings (Benke et al., 2023; Zeng et al., 2023) The pandemic experience appears to be cognitively processed in a traumatic frame, which makes COVID-related stimuli perceived as threatening and anxiogenic. Conversely, negative stimuli unrelated to the pandemic elicit general negative emotions, which prompt the activation of automatic and related aspects of danger and threat response, thereby facilitating the pop-out effect. Further studies should explore these insights.

Despite the many insights that this study offers, some limitations should be highlighted. The first limitation concerns the control of some psychological variables. Although we monitored the levels of PTSD-related to the COVID-19 pandemic in a retrospective-current double evaluation and anxiety as trait levels, future studies should be considering the current self-reported activation according to the different sections of the task to provide information about the arousal and emotional activation that the participants experience with respect to each emotional valence. Another limitation is the absence of a positive valence in the stimuli of the Flicker Task (Forte et al., 2021). The neutral valence may be considered as a control dimension; however, it would be interesting to examine the extent to which bottom-up and top-down processes are involved and differ from those elicited by threatening stimuli (e.g., negative non-COVID), anxious stimuli (e.g., COVID-related stimuli) and positive stimuli (arousal-activating but with positive feeling from a physiological and cognitive perspective).

## 5 Conclusion

Our findings suggest that the pandemic experience may have altered the typical attentional processes and their underlying mechanisms for stimuli related to the pandemic. The correlation between high HRV and poorer performance in managing central changes indicates a complex interaction between autonomic functions and cognitive processes during the elaboration of emotional stimuli, particularly in the context of traumatic pandemic experiences. In conclusion, the results of this study provide further support to the hypothesis that the experience of the pandemic affects attentional processes (Favieri et al., 2021b; Forte et al., 2024). However, it also suggests that there has been a shift in the perception of pandemic-related stimuli, whereby they are now perceived as a potential source of survival rather than a threat. This shift allows for the interference of top-down and bottom-up processes in attentional processing. Further investigations are needed to understand how pandemic-related stressors might have shaped attentional dynamics and autonomic responses, potentially influencing broader cognitive functions and behaviors. From an applicational perspective, especially in the clinical field, understanding the impact of emotionally salient stimuli on cognitive functions such as attentional processes, may prove relevant insight. In particular, it would help in developing procedure and intervention aimed at reducing the impact of highly emotionally charged events. Furthermore, it would facilitate the adoption of attentional paradigms t that could potentially mitigate the influence of such significant stimuli.

## Data Availability

The raw data supporting the conclusions of this article will be made available by the authors, without undue reservation.

## Footnotes

^1^ https://osf.io/zm9s5/?view_only=6fdd05e6b4a2447daaff89f607c70f17
